# 2-Meth­oxy-1-(2-meth­oxy-4-nitro­naphthalen-1-yl)-6-nitro­naphthalene

**DOI:** 10.1107/S1600536813008660

**Published:** 2013-04-10

**Authors:** Boontana Wannalerse, Wilasinee Pannil, Jaruwan Loriang, Thawatchai Tuntulani, Tanwawan Duangthongyou

**Affiliations:** a Department of Chemistry and Center of Excellence for Innovation in Chemistry, Faculty of Science, Kasetsart University, Bangkok 10903, Thailand; b Supramolecular Chemistry Research Unit, Department of Chemistry, Faculty of Science, Chulalongkorn University, Bangkok 10330, Thailand; c Department of Chemistry, Faculty of Science, Kasetsart University, Bangkok 10903, Thailand

## Abstract

In the title compound, C_22_H_16_N_2_O_6_, the naphthalene ring systems form a dihedral angle of 65.2 (1)°. Two O atoms of one of the nitro groups are disordered over two sets of sites with occupancy factors of 0.586 (15) and 0.414 (15). Weak C—H⋯O inter­molecular inter­actions are present, forming a ladder like structure along the *a* axis.

## Related literature

For related structures, see: Thorup *et al.* (2006 ▶,) ; Thoss *et al.* (2009 ▶); Ge & Li (2009 ▶).
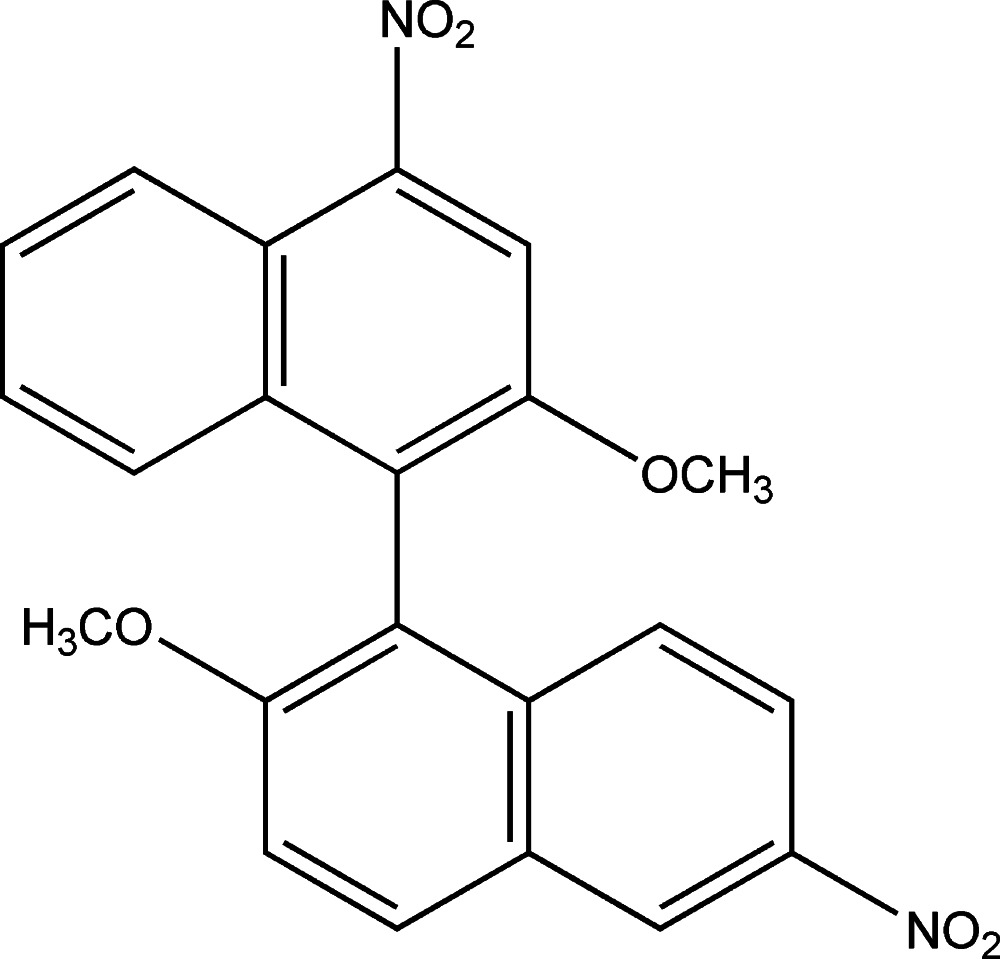


## Experimental

### 

#### Crystal data

C_22_H_16_N_2_O_6_
*M* *_r_* = 404.37
Orthorhombic, 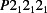

*a* = 7.095 (16) Å
*b* = 15.14 (4) Å
*c* = 17.86 (5) Å
*V* = 1918 (9) Å^3^
*Z* = 4
Mo *K*α radiation
μ = 0.10 mm^−1^
*T* = 296 K
0.36 × 0.16 × 0.14 mm

#### Data collection

Bruker APEXII CCD diffractometer
3043 measured reflections
1881 independent reflections
1440 reflections with *I* > 2σ(*I*)
*R*_int_ = 0.023

#### Refinement

*R*[*F* ^2^ > 2σ(*F* ^2^)] = 0.035
*wR*(*F* ^2^) = 0.090
*S* = 1.01
1881 reflections
292 parameters
H-atom parameters constrained
Δρ_max_ = 0.10 e Å^−3^
Δρ_min_ = −0.16 e Å^−3^

### 

Data collection: *APEX2* (Bruker, 2011 ▶); cell refinement: *SAINT* (Bruker, 2011 ▶); data reduction: *SAINT*; program(s) used to solve structure: *SHELXS97* (Sheldrick, 2008 ▶); program(s) used to refine structure: *SHELXL97* (Sheldrick, 2008 ▶); molecular graphics: *SHELXTL* (Sheldrick, 2008 ▶); software used to prepare material for publication: *SHELXTL*.

## Supplementary Material

Crystal structure: contains datablock(s) I, global. DOI: 10.1107/S1600536813008660/lr2099sup1.cif

Structure factors: contains datablock(s) I. DOI: 10.1107/S1600536813008660/lr2099Isup2.hkl

Supplementary material file. DOI: 10.1107/S1600536813008660/lr2099Isup3.cml

Additional supplementary materials:  crystallographic information; 3D view; checkCIF report

## Figures and Tables

**Table 1:** Hydrogen-bond geometry (Å, °)

*D*—H⋯*A*	*D*—H	H⋯*A*	*D*⋯*A*	*D*—H⋯*A*
C17—H17⋯O2^i^	0.93	2.59	3.361 (10)	140

Symmetry code: (i) 
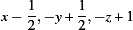
.

**Fig. 1.**
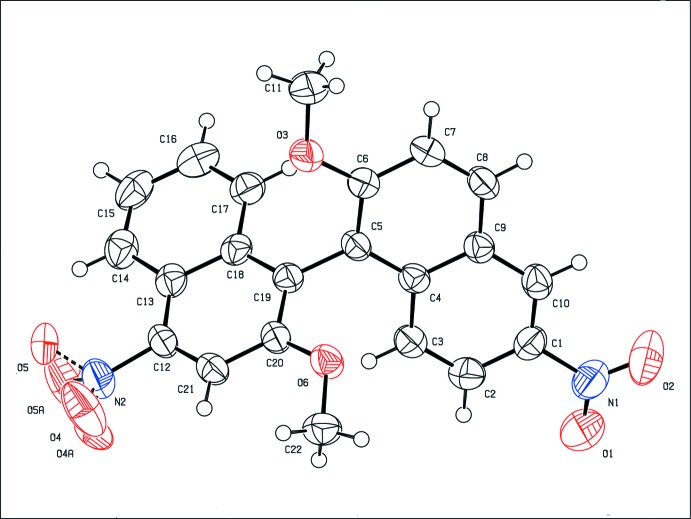
Molecular structure of the title compound (arbitrary spheres for the H atoms). Displacement ellipsoids are drawn at the 50% probability level.

**Fig. 2.**
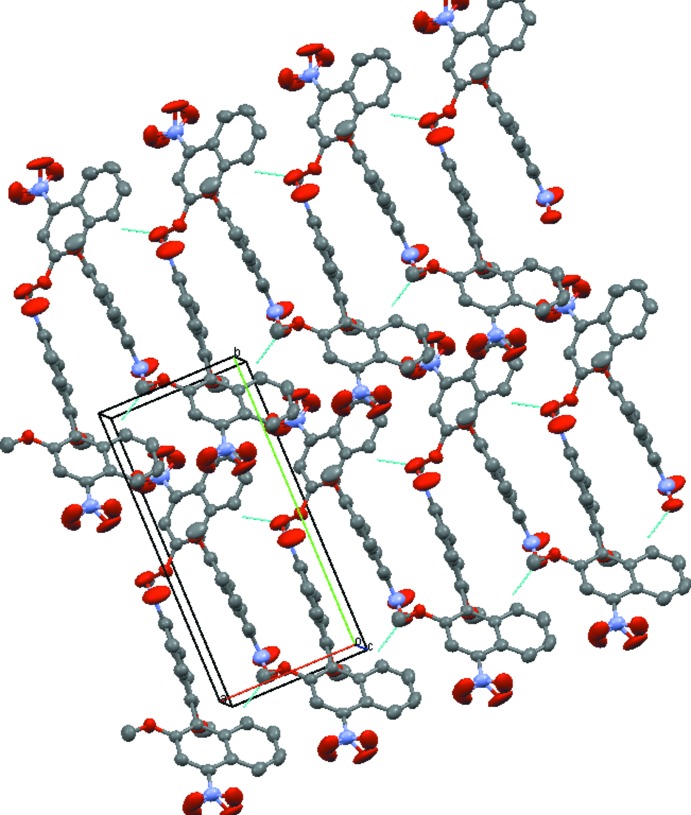
A packing view of the compound (dash lines represent weak C—H···O interaction).

## supplementary crystallographic information

### Comment

The molecule of the title compound, C_22_H_16_N_2_O_6_, contains two naphthalene rings forming a dihedral angle of 65.2 (1)° between them. The molecular structure is shown in Fig.1. The crystal structure is stabilized by weak C17—H17···O2 intermolecular interactions forming a ladder like structure, along the *a* axis (Fig. 2).

### Experimental

The (*S*)-2,2'-dimethoxy-1,1'-binaphthalene (0.5 g,1.98 mmol) was stirred in CH_3_CN(10 ml). Nitric acid (0.4 ml) in glacial acetic acid (5 ml) was added dropwise in 10 minutes. The mixture was then stirred at 70°C for 16 h after pouring into the water (50 ml) and extracted with dichloromethane (3x50 ml). The organic layer was washed with 1*M* KOH, brine and dried over Na_2_SO_4_. The mixture was filtrated and evaporated of the solvent afforded a yellow solid,then purified by column chromatography using dichloromethane: hexane = 7:3 as an eluent. Brown crystal were obtained from a mixture of dichloromethane:hexane.

### Refinement

All H atoms of the compound were placed in calculated positions with C—H = 0.93 Å (aromatic hydrogen) and 0.96 Å (methyl hydrogen) and included in the cycles of refinement using a riding model with *U*_iso_(H) = 1.2 *U*_eq_(C) and *U*_iso_(H) = 1.5 *U*_eq_(C) ,for aromatic and methyl hydrogens respectively. The resulting Flack's parameter value was meaningless and no absolute structure could be fixed and the Fiedel's pairs were merged in the last refinement.

### Crystal data

**Table:**

C_22_H_16_N_2_O_6_	*F*(000) = 840
*M**_r_* = 404.37	*D*_x_ = 1.400 Mg m^−^^3^
Orthorhombic, *P*2_1_2_1_2_1_	Mo *K*α radiation, λ = 0.71073 Å
Hall symbol: P 2ac 2ab	Cell parameters from 1608 reflections
*a* = 7.095 (16) Å	θ = 2.7–18.9°
*b* = 15.14 (4) Å	µ = 0.10 mm^−^^1^
*c* = 17.86 (5) Å	*T* = 296 K
*V* = 1918 (9) Å^3^	Bar, brown
*Z* = 4	0.36 × 0.16 × 0.14 mm

### Data collection

**Table:**

Bruker APEXII CCD diffractometer	1440 reflections with *I* > 2σ(*I*)
Radiation source: fine-focus sealed tube	*R*_int_ = 0.023
Graphite monochromator	θ_max_ = 24.8°, θ_min_ = 2.9°
φ and ω scans	*h* = −6→8
3043 measured reflections	*k* = 0→17
1881 independent reflections	*l* = 0→20

### Refinement

**Table:**

Refinement on *F*^2^	Primary atom site location: structure-invariant direct methods
Least-squares matrix: full	Secondary atom site location: difference Fourier map
*R*[*F*^2^ > 2σ(*F*^2^)] = 0.035	Hydrogen site location: inferred from neighbouring sites
*wR*(*F*^2^) = 0.090	H-atom parameters constrained
*S* = 1.01	*w* = 1/[σ^2^(*F*_o_^2^) + (0.0534*P*)^2^ + 0.0581*P*] where *P* = (*F*_o_^2^ + 2*F*_c_^2^)/3
1881 reflections	(Δ/σ)_max_ < 0.001
292 parameters	Δρ_max_ = 0.10 e Å^−^^3^
0 restraints	Δρ_min_ = −0.16 e Å^−^^3^

### Special details

**Table:**

Geometry. All esds (except the esd in the dihedral angle between two l.s. planes) are estimated using the full covariance matrix. The cell esds are taken into account individually in the estimation of esds in distances, angles and torsion angles; correlations between esds in cell parameters are only used when they are defined by crystal symmetry. An approximate (isotropic) treatment of cell esds is used for estimating esds involving l.s. planes.
Refinement. Refinement of F^2^ against ALL reflections. The weighted R-factor wR and goodness of fit S are based on F^2^, conventional R-factors R are based on F, with F set to zero for negative F^2^. The threshold expression of F^2^ > 2sigma(F^2^) is used only for calculating R-factors(gt) etc. and is not relevant to the choice of reflections for refinement. R-factors based on F^2^ are statistically about twice as large as those based on F, and R- factors based on ALL data will be even larger.

### Fractional atomic coordinates and isotropic or equivalent isotropic displacement parameters (Å^2^)

**Table:**

	*x*	*y*	*z*	*U*_iso_*/*U*_eq_	Occ. (<1)
O1	0.1345 (6)	0.40153 (18)	0.28224 (16)	0.1209 (13)	
O2	0.1746 (4)	0.46589 (15)	0.38886 (16)	0.0921 (8)	
O3	0.2362 (3)	−0.03073 (11)	0.59542 (10)	0.0567 (6)	
O6	0.5329 (3)	0.02923 (12)	0.40128 (11)	0.0520 (5)	
N1	0.1607 (4)	0.39888 (18)	0.35029 (19)	0.0688 (8)	
N2	0.3170 (5)	−0.25427 (17)	0.30186 (17)	0.0626 (7)	
C1	0.1670 (4)	0.31102 (18)	0.38691 (17)	0.0480 (7)	
C2	0.1735 (4)	0.23483 (18)	0.34021 (16)	0.0515 (7)	
H2	0.1702	0.2405	0.2884	0.062*	
C3	0.1849 (4)	0.15274 (19)	0.37294 (15)	0.0484 (7)	
H3	0.1889	0.1028	0.3426	0.058*	
C4	0.1908 (4)	0.14237 (16)	0.45239 (15)	0.0393 (6)	
C5	0.2108 (4)	0.05732 (16)	0.48721 (14)	0.0405 (6)	
C6	0.2104 (4)	0.05224 (16)	0.56536 (14)	0.0437 (7)	
C7	0.1857 (4)	0.12987 (18)	0.61018 (16)	0.0496 (7)	
H7	0.1817	0.1252	0.6621	0.059*	
C8	0.1679 (4)	0.21108 (18)	0.57691 (15)	0.0478 (7)	
H8	0.1519	0.2608	0.6068	0.057*	
C9	0.1735 (4)	0.22098 (16)	0.49791 (15)	0.0411 (7)	
C10	0.1630 (4)	0.30550 (17)	0.46272 (17)	0.0474 (7)	
H10	0.1534	0.3564	0.4916	0.057*	
C11	0.2143 (6)	−0.0425 (2)	0.67474 (16)	0.0815 (11)	
H11A	0.0950	−0.0188	0.6902	0.122*	
H11B	0.3140	−0.0122	0.7005	0.122*	
H11C	0.2192	−0.1043	0.6865	0.122*	
C12	0.2779 (4)	−0.17661 (17)	0.35106 (14)	0.0447 (7)	
C13	0.1069 (4)	−0.17088 (17)	0.39423 (15)	0.0442 (7)	
C14	−0.0412 (4)	−0.23552 (19)	0.39645 (17)	0.0565 (8)	
H14	−0.0313	−0.2861	0.3672	0.068*	
C15	−0.1982 (5)	−0.2240 (2)	0.44130 (18)	0.0622 (9)	
H15	−0.2907	−0.2675	0.4428	0.075*	
C16	−0.2194 (5)	−0.1468 (2)	0.48479 (17)	0.0606 (8)	
H16	−0.3267	−0.1390	0.5140	0.073*	
C17	−0.0808 (4)	−0.08277 (19)	0.48406 (15)	0.0493 (7)	
H17	−0.0969	−0.0322	0.5129	0.059*	
C18	0.0887 (4)	−0.09202 (16)	0.43966 (15)	0.0410 (6)	
C19	0.2341 (4)	−0.02545 (16)	0.44096 (13)	0.0396 (6)	
C20	0.3965 (4)	−0.03611 (16)	0.39655 (15)	0.0414 (6)	
C21	0.4176 (4)	−0.11250 (16)	0.35163 (15)	0.0445 (7)	
H21	0.5252	−0.1197	0.3225	0.053*	
C22	0.6813 (4)	0.0311 (2)	0.34571 (16)	0.0582 (8)	
H22A	0.6268	0.0373	0.2968	0.087*	
H22B	0.7521	−0.0228	0.3481	0.087*	
H22C	0.7634	0.0802	0.3554	0.087*	
O4A	0.448 (2)	−0.2544 (8)	0.2597 (5)	0.093 (3)	0.586 (15)
O5A	0.1822 (7)	−0.2895 (6)	0.2681 (5)	0.099 (4)	0.586 (15)
O4B	0.479 (2)	−0.2756 (12)	0.2933 (11)	0.098 (5)	0.414 (15)
O5B	0.250 (2)	−0.3295 (4)	0.3232 (8)	0.114 (6)	0.414 (15)

### Atomic displacement parameters (Å^2^)

**Table:**

	*U*^11^	*U*^22^	*U*^33^	*U*^12^	*U*^13^	*U*^23^
O1	0.222 (4)	0.0738 (17)	0.0670 (17)	0.010 (2)	0.024 (2)	0.0264 (15)
O2	0.118 (2)	0.0473 (14)	0.111 (2)	−0.0124 (14)	−0.0229 (18)	0.0075 (15)
O3	0.0807 (14)	0.0467 (11)	0.0425 (10)	0.0087 (11)	0.0065 (11)	0.0015 (10)
O6	0.0450 (10)	0.0511 (11)	0.0599 (12)	−0.0070 (9)	0.0132 (10)	−0.0137 (11)
N1	0.0765 (19)	0.0542 (17)	0.076 (2)	−0.0037 (15)	0.0094 (17)	0.0106 (17)
N2	0.078 (2)	0.0498 (17)	0.0600 (19)	0.0029 (17)	−0.0004 (18)	−0.0139 (15)
C1	0.0424 (15)	0.0427 (15)	0.059 (2)	0.0005 (14)	0.0053 (14)	0.0083 (14)
C2	0.0532 (17)	0.0575 (18)	0.0437 (17)	0.0017 (16)	0.0024 (14)	0.0016 (16)
C3	0.0540 (18)	0.0464 (16)	0.0449 (16)	0.0049 (16)	0.0043 (15)	−0.0033 (13)
C4	0.0341 (14)	0.0427 (15)	0.0411 (15)	0.0013 (13)	0.0030 (12)	−0.0026 (13)
C5	0.0412 (15)	0.0388 (14)	0.0414 (14)	0.0023 (13)	0.0053 (12)	−0.0060 (12)
C6	0.0462 (16)	0.0440 (15)	0.0409 (15)	0.0012 (14)	0.0035 (13)	−0.0002 (13)
C7	0.0568 (17)	0.0544 (18)	0.0375 (16)	0.0021 (15)	0.0052 (15)	−0.0068 (14)
C8	0.0513 (16)	0.0459 (16)	0.0462 (17)	0.0036 (14)	0.0055 (14)	−0.0123 (14)
C9	0.0368 (14)	0.0408 (14)	0.0457 (16)	0.0006 (14)	0.0026 (12)	−0.0035 (13)
C10	0.0407 (15)	0.0413 (15)	0.0601 (19)	0.0005 (13)	0.0069 (14)	−0.0085 (15)
C11	0.136 (3)	0.0622 (19)	0.0467 (18)	0.007 (2)	0.020 (2)	0.0107 (16)
C12	0.0596 (18)	0.0376 (14)	0.0368 (14)	0.0100 (15)	−0.0055 (14)	−0.0056 (13)
C13	0.0510 (16)	0.0395 (14)	0.0420 (16)	0.0047 (14)	−0.0071 (13)	0.0053 (14)
C14	0.069 (2)	0.0482 (16)	0.053 (2)	−0.0082 (16)	−0.0136 (17)	0.0005 (16)
C15	0.063 (2)	0.0585 (19)	0.065 (2)	−0.0234 (18)	−0.0104 (18)	0.0144 (17)
C16	0.0518 (19)	0.072 (2)	0.0583 (19)	−0.0074 (18)	0.0034 (16)	0.0146 (18)
C17	0.0500 (16)	0.0501 (15)	0.0479 (17)	0.0034 (16)	0.0036 (14)	0.0059 (15)
C18	0.0470 (15)	0.0383 (14)	0.0376 (14)	0.0025 (14)	−0.0040 (13)	0.0072 (13)
C19	0.0446 (15)	0.0393 (14)	0.0349 (14)	0.0036 (13)	0.0002 (13)	0.0014 (12)
C20	0.0461 (15)	0.0379 (14)	0.0403 (15)	0.0036 (13)	−0.0008 (13)	−0.0042 (14)
C21	0.0471 (16)	0.0456 (15)	0.0408 (16)	0.0078 (14)	0.0020 (13)	−0.0045 (14)
C22	0.0557 (18)	0.0656 (19)	0.0532 (17)	−0.0046 (17)	0.0131 (16)	−0.0008 (17)
O4A	0.132 (9)	0.072 (5)	0.075 (5)	−0.014 (5)	0.053 (6)	−0.031 (4)
O5A	0.085 (3)	0.096 (6)	0.117 (7)	−0.003 (3)	−0.020 (3)	−0.061 (5)
O4B	0.073 (5)	0.095 (10)	0.125 (13)	0.021 (6)	0.005 (8)	−0.052 (9)
O5B	0.194 (12)	0.035 (4)	0.112 (9)	−0.022 (5)	0.071 (9)	−0.015 (4)

### Geometric parameters (Å, º)

**Table:**

O1—N1	1.230 (5)	C8—H8	0.9300
O2—N1	1.230 (4)	C9—C10	1.428 (5)
O3—C6	1.378 (4)	C10—H10	0.9300
O3—C11	1.436 (5)	C11—H11A	0.9600
O6—C20	1.386 (4)	C11—H11B	0.9600
O6—C22	1.447 (4)	C11—H11C	0.9600
N1—C1	1.483 (5)	C12—C21	1.387 (5)
N2—O4A	1.193 (12)	C12—C13	1.440 (5)
N2—O4B	1.206 (17)	C13—C14	1.436 (5)
N2—O5A	1.250 (5)	C13—C18	1.449 (5)
N2—O5B	1.292 (7)	C14—C15	1.383 (5)
N2—C12	1.494 (4)	C14—H14	0.9300
C1—C10	1.357 (5)	C15—C16	1.412 (5)
C1—C2	1.424 (5)	C15—H15	0.9300
C2—C3	1.376 (5)	C16—C17	1.381 (5)
C2—H2	0.9300	C16—H16	0.9300
C3—C4	1.428 (5)	C17—C18	1.448 (5)
C3—H3	0.9300	C17—H17	0.9300
C4—C5	1.437 (5)	C18—C19	1.442 (4)
C4—C9	1.447 (5)	C19—C20	1.408 (4)
C5—C6	1.398 (5)	C20—C21	1.416 (4)
C5—C19	1.510 (5)	C21—H21	0.9300
C6—C7	1.433 (5)	C22—H22A	0.9600
C7—C8	1.371 (5)	C22—H22B	0.9600
C7—H7	0.9300	C22—H22C	0.9600
C8—C9	1.419 (5)		
			
C6—O3—C11	118.9 (2)	C9—C10—H10	120.2
C20—O6—C22	118.7 (2)	O3—C11—H11A	109.5
O1—N1—O2	122.6 (3)	O3—C11—H11B	109.5
O1—N1—C1	118.0 (3)	H11A—C11—H11B	109.5
O2—N1—C1	119.4 (3)	O3—C11—H11C	109.5
O4A—N2—O4B	34.8 (7)	H11A—C11—H11C	109.5
O4A—N2—O5A	106.8 (8)	H11B—C11—H11C	109.5
O4B—N2—O5A	123.8 (9)	C21—C12—C13	123.8 (3)
O4A—N2—O5B	118.1 (8)	C21—C12—N2	115.0 (3)
O4B—N2—O5B	98.9 (13)	C13—C12—N2	121.3 (3)
O5A—N2—O5B	58.9 (5)	C14—C13—C12	126.2 (3)
O4A—N2—C12	121.1 (6)	C14—C13—C18	118.7 (3)
O4B—N2—C12	117.6 (8)	C12—C13—C18	115.1 (2)
O5A—N2—C12	118.5 (4)	C15—C14—C13	121.3 (3)
O5B—N2—C12	116.9 (4)	C15—C14—H14	119.4
C10—C1—C2	122.4 (3)	C13—C14—H14	119.4
C10—C1—N1	119.6 (3)	C14—C15—C16	120.6 (3)
C2—C1—N1	118.0 (3)	C14—C15—H15	119.7
C3—C2—C1	119.0 (3)	C16—C15—H15	119.7
C3—C2—H2	120.5	C17—C16—C15	120.0 (3)
C1—C2—H2	120.5	C17—C16—H16	120.0
C2—C3—C4	121.6 (3)	C15—C16—H16	120.0
C2—C3—H3	119.2	C16—C17—C18	121.9 (3)
C4—C3—H3	119.2	C16—C17—H17	119.0
C3—C4—C5	122.1 (2)	C18—C17—H17	119.0
C3—C4—C9	117.7 (3)	C19—C18—C17	121.2 (3)
C5—C4—C9	120.2 (3)	C19—C18—C13	121.4 (3)
C6—C5—C4	118.8 (2)	C17—C18—C13	117.4 (2)
C6—C5—C19	120.0 (2)	C20—C19—C18	119.7 (3)
C4—C5—C19	121.2 (3)	C20—C19—C5	119.5 (2)
O3—C6—C5	116.0 (2)	C18—C19—C5	120.7 (2)
O3—C6—C7	123.1 (3)	O6—C20—C19	117.1 (2)
C5—C6—C7	120.9 (3)	O6—C20—C21	122.9 (3)
C8—C7—C6	120.3 (3)	C19—C20—C21	120.0 (2)
C8—C7—H7	119.8	C12—C21—C20	120.0 (3)
C6—C7—H7	119.8	C12—C21—H21	120.0
C7—C8—C9	121.5 (2)	C20—C21—H21	120.0
C7—C8—H8	119.2	O6—C22—H22A	109.5
C9—C8—H8	119.2	O6—C22—H22B	109.5
C8—C9—C10	122.1 (2)	H22A—C22—H22B	109.5
C8—C9—C4	118.3 (3)	O6—C22—H22C	109.5
C10—C9—C4	119.6 (3)	H22A—C22—H22C	109.5
C1—C10—C9	119.6 (3)	H22B—C22—H22C	109.5
C1—C10—H10	120.2		
			
O1—N1—C1—C10	−168.7 (3)	O4B—N2—C12—C13	−148.8 (11)
O2—N1—C1—C10	8.6 (5)	O5A—N2—C12—C13	36.0 (7)
O1—N1—C1—C2	10.1 (5)	O5B—N2—C12—C13	−31.5 (11)
O2—N1—C1—C2	−172.6 (3)	C21—C12—C13—C14	−179.3 (3)
C10—C1—C2—C3	−3.1 (4)	N2—C12—C13—C14	0.5 (4)
N1—C1—C2—C3	178.2 (3)	C21—C12—C13—C18	0.0 (4)
C1—C2—C3—C4	−0.2 (4)	N2—C12—C13—C18	179.7 (2)
C2—C3—C4—C5	−177.3 (3)	C12—C13—C14—C15	178.6 (3)
C2—C3—C4—C9	3.5 (4)	C18—C13—C14—C15	−0.6 (4)
C3—C4—C5—C6	−178.3 (3)	C13—C14—C15—C16	1.7 (4)
C9—C4—C5—C6	0.9 (4)	C14—C15—C16—C17	−1.4 (5)
C3—C4—C5—C19	2.3 (4)	C15—C16—C17—C18	−0.1 (4)
C9—C4—C5—C19	−178.4 (2)	C16—C17—C18—C19	−178.5 (3)
C11—O3—C6—C5	−172.4 (3)	C16—C17—C18—C13	1.2 (4)
C11—O3—C6—C7	8.0 (4)	C14—C13—C18—C19	178.8 (2)
C4—C5—C6—O3	−178.0 (2)	C12—C13—C18—C19	−0.5 (3)
C19—C5—C6—O3	1.4 (4)	C14—C13—C18—C17	−0.9 (3)
C4—C5—C6—C7	1.6 (4)	C12—C13—C18—C17	179.8 (2)
C19—C5—C6—C7	−179.1 (2)	C17—C18—C19—C20	−179.4 (2)
O3—C6—C7—C8	177.5 (3)	C13—C18—C19—C20	0.9 (4)
C5—C6—C7—C8	−2.1 (4)	C17—C18—C19—C5	−1.8 (4)
C6—C7—C8—C9	0.0 (5)	C13—C18—C19—C5	178.6 (2)
C7—C8—C9—C10	−177.4 (3)	C6—C5—C19—C20	−114.4 (3)
C7—C8—C9—C4	2.5 (4)	C4—C5—C19—C20	64.9 (3)
C3—C4—C9—C8	176.4 (2)	C6—C5—C19—C18	68.0 (3)
C5—C4—C9—C8	−2.9 (4)	C4—C5—C19—C18	−112.7 (3)
C3—C4—C9—C10	−3.8 (4)	C22—O6—C20—C19	−166.9 (2)
C5—C4—C9—C10	176.9 (3)	C22—O6—C20—C21	15.2 (4)
C2—C1—C10—C9	2.7 (5)	C18—C19—C20—O6	−178.8 (2)
N1—C1—C10—C9	−178.5 (2)	C5—C19—C20—O6	3.5 (3)
C8—C9—C10—C1	−179.3 (3)	C18—C19—C20—C21	−0.8 (4)
C4—C9—C10—C1	0.8 (4)	C5—C19—C20—C21	−178.5 (2)
O4A—N2—C12—C21	−9.0 (9)	C13—C12—C21—C20	0.1 (4)
O4B—N2—C12—C21	30.9 (12)	N2—C12—C21—C20	−179.6 (2)
O5A—N2—C12—C21	−144.3 (7)	O6—C20—C21—C12	178.2 (2)
O5B—N2—C12—C21	148.3 (10)	C19—C20—C21—C12	0.3 (4)
O4A—N2—C12—C13	171.3 (8)		

### Hydrogen-bond geometry (Å, º)

**Table:**

*D*—H···*A*	*D*—H	H···*A*	*D*···*A*	*D*—H···*A*
C17—H17···O2^i^	0.93	2.59	3.361 (10)	140

Symmetry code: (i) *x*−1/2, −*y*+1/2, −*z*+1.
